# Intact blood pressure, but not sympathetic, responsiveness to sympathoexcitatory stimuli in a patient with unilateral carotid body resection

**DOI:** 10.14814/phy2.13212

**Published:** 2017-03-31

**Authors:** Kathryn F. Larson, Jacqueline K. Limberg, Sarah E. Baker, Michael J. Joyner, Timothy B. Curry

**Affiliations:** ^1^ Department of AnesthesiologyMayo ClinicRochesterMinnesota

**Keywords:** Baroreflex sensitivity, carotid chemoreceptor, hypoxic ventilatory response, muscle sympathetic nerve activity

## Abstract

Despite rapidly growing interest in the therapeutic resection of the carotid body (CB) chemoreceptors, few physiologic studies exist on the consequences of unilateral CB resection. We present a case of an otherwise healthy postmenopausal female who underwent unilateral CB resection for a paraganglioma. Approximately 4 years postoperatively, she underwent analysis of her sympathetic and hemodynamic responses to hypoxia, lower body negative pressure, cold pressor test (CPT), and ischemic hand grip exercise and postexercise ischemia (IHE/PEI). Hypoxic ventilatory response and baroreflex sensitivity were relatively normal. Hemodynamic responses to IHE/PEI and CPT showed characteristic increases in cardiac output (from 3.9 L/min to 5.2 L/min [IHE/PEI] and 4.9 L/min [CPT]) and blood pressure (from 126/72 mmHg to 161/87 mmHg [IHE/PEI] and 171/93 mmHg [CPT]). However, muscle sympathetic nerve activity (microneurography of the peroneal nerve) decreased from baseline during IHE/PEI and CPT (burst incidence nadir of 45% and 40% of baseline, respectively) and there was no observable change in total peripheral resistance (from 24 mmHg*min/L to 22 mmHg*min/L [IHE/PEI] and 25 mmHg*min/L [CPT]). These findings illustrate intact blood pressure responsiveness despite attenuated sympathoexcitation, possibly due to an increase in cardiac output and/or adaptive inhibitory effect of the baroreflex on peripheral sympathetic activity.

## Introduction

The carotid bodies (CBs) are sensory organs located bilaterally at the bifurcation of the common carotid arteries. Hypoxia is sensed by the type 1 glomus cell within the carotid body which transmits this information to the brainstem via the afferent sensory fibers of cranial nerve IX (glossopharyngeus). In turn, this signal reflexively stimulates centrally mediated sympathetic activity. There is growing evidence that the carotid bodies are responsible, at least in part, for tonic sympathetic nervous system activity in multiple disease states in addition to stimulated increases during routine physiologic stressors such as exercise (Paton et al. 2013). These diseases include but are not limited to congestive heart failure, sleep disordered breathing, and hypertension (Paton et al. 2013; Narkiewicz et al. 2016). As such, the carotid bodies provide a potential therapeutic target for modulation in disease states associated with increased sympathetic activity.

Over the past few years, the literature regarding therapeutic CB resection has grown. Patients who undergo CB resection provide a unique and timely in vivo opportunity to study the relative contribution of the CBs to sympathetic output. There are multiple studies evaluating the physiological consequences of bilateral CB resection in the literature. Bilateral CB resection results in a significantly decreased ventilatory response to hypoxia (HVR) (Honda et al. 1979). Studies evaluating baroreceptor function suggest an increase in blood pressure (BP) variability and occasional complete baroreflex failure following bilateral CB resection (Timmers et al. 2003; Fudim et al. 2015). In comparison, only a handful of studies have evaluated the physiological consequences of unilateral CB resection. Evidence suggests the HVR is maintained in unilateral resection, yet, data on changes in BP and baroreflex sensitivity (BRS) are mixed (Dehn and Angell‐James 1987; Timmers et al. 2003; Fudim et al. 2015). Overall, data regarding physiological consequences of unilateral CB resection are lacking in healthy humans.

We present the case of an otherwise healthy female who underwent unilateral CB resection for a paraganglioma. Subsequently, she underwent an investigation of her remaining CB function and sensitivity to sympathetic and hemodynamic stressors. Specifically, we investigated measures of sympathetic nervous system responsiveness to hypoxia, traditional sympathetic stimuli (cold pressor test, ischemic handgrip), and lower body negative pressure.

## Case Report

**Figure 1:**
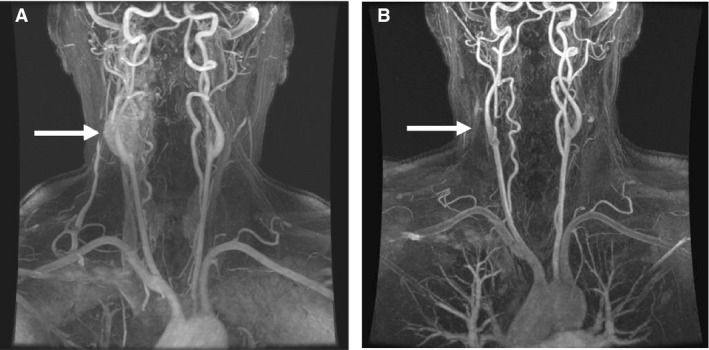
Magnetic resonance angiography scans pre‐(A) and post‐(B) paraganglioma resection. Imaging revealed the presence of bilateral carotid body paragangliomas, 2.7 cm in diameter on the right, and 0.6 cm in diameter on the left. The patient underwent uneventful elective unilateral resection of the right paraganglioma. (A) Preoperative imaging. (B) Postoperative imaging.

A 54‐year‐old postmenopausal female with a past medical history of osteoarthritis was referred to our institution for a mass associated with her carotid artery. Imaging revealed the presence of bilateral carotid body paragangliomas, 2.7 cm in diameter on the right and 0.6 cm in diameter on the left (see Fig. 1). She denied symptoms of orthostasis, exercise intolerance, dyspnea, or dysphagia. She had no history of hypertension or respiratory disease, including sleep disordered breathing. An extensive neurohormonal workup revealed no abnormal elevations in serum or urinary catecholamines. She underwent uneventful elective unilateral resection of the right paraganglioma. Operative notes reveal multiple nerves were identified and preserved, including the spinal accessory, hypoglossal, and vagus nerves. Histologically, the paraganglioma arose from glomus cells within the carotid body. The tumor was completely excised with negative margins.

Approximately 4 years postoperatively, she was studied in our laboratory to evaluate chemoreceptor sensitivity, sympathetic activity, and BP regulation. All procedures were approved by the Institutional Review Board at Mayo Clinic. Informed consent was obtained in writing before study enrollment and testing. The study conformed to the Declaration of Helsinki.

Initially, a normocapnic hypoxic ventilatory response (HVR) test was completed to determine CB chemosensitivity to hypoxia (Limberg et al. 2016). Afterwards, she performed isometric hand grip exercise and postexercise ischemia (IHE/PEI) and cold pressor test (CPT) as sympathetic stimuli (Victor et al. 1987). Measures of heart rate (HR), blood pressure (BP), minute ventilation, and muscle sympathetic nerve activity (MSNA, microneurography of the peroneal nerve) were obtained during these tests (Limberg et al. 2015). Measures of HR variability (HRV), cardiac output (CO), and total peripheral resistance (TPR) were calculated using three lead electrocardiography, finger photoplethysmography, and off‐line analysis with custom software (WinCPRS, Version 1.163, Absolute Aliens Oy, Turku, Finland) (Limberg et al. 2016). Baroreflex sensitivity (BRS) was measured via trials of lower body negative pressure (LBNP, for simulation of venous pooling) (Somers et al. 1991; Thompson et al. 1999). The methodologies for these interventions have been described elsewhere (Johnson et al. 2014; Limberg et al. 2016). Typical physiologic responses in anatomically intact humans would be increased sympathetic output (i.e., increased HR, BP, ventilatory rate, and increased MSNA) during stressors such as IHE/PEI, CPT, and LBNP.

During graded trials of increasing levels of hypoxia and stable normocapnia, the slope of the regression line between S_p_O_2_ and minute ventilation (a measure of CB sensitivity to hypoxia, HVR) was measured as −0.23 L/min/% (healthy range: −0.19 to −0.93 L/min/% (Limberg et al. 2016)). MSNA burst area was inversely correlated with S_p_O_2_ (*r* = −0.48).

Application of LBNP showed increases in HR as LBNP increased; similarly, mean arterial pressure increased (96–102 mmHg) as LBNP increased. There was an eventual drop in mean arterial pressure (−14 mmHg) at the highest level of LBNP (−45 mmHg) where HR continued to increase. The rise in MAP and HR with increasingly negative levels of LBNP is consistent with previous studies in anatomically intact individuals as well as denervated dogs (Melcher and Donald 1981; Hachiya et al. 2012). This indicates that our patient's BRS was intact. Of note, the patient reported a subjective “hot flash” during this time. This may have represented presyncope or a hormonal hot flash triggered by LBNP that resulted in significant peripheral vasodilation, and a decrease in BP.

**Figure 2:**
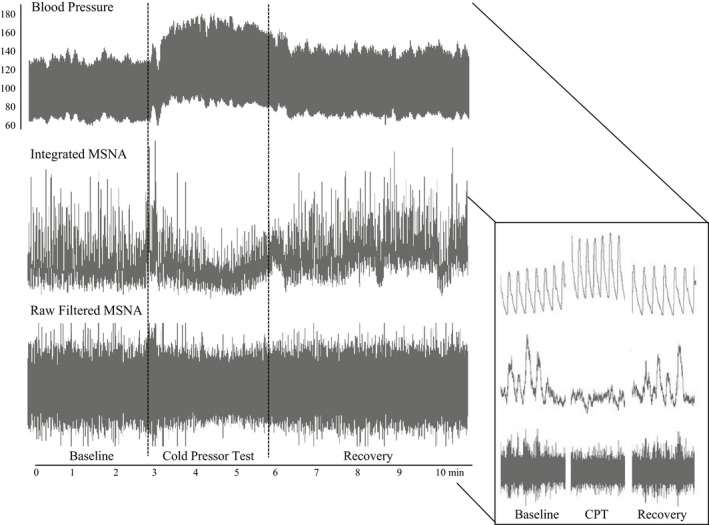
Compressed data from the patient's cold pressor test. Data are shown from quiet baseline prior to cold pressor test, 3 min during cold pressor test, and 3 min of recovery after the cold pressor test. MSNA, muscle sympathetic nerve activity.

During IHE/PEI and CPT, the patient's MSNA measured in burst frequency (bursts/min), burst incidence (bursts/100 heart beats), and burst area were obtained. During IHE/PEI and CPT, burst frequency, burst incidence, and burst area uncharacteristically decreased from baseline values (Fig. 2). In contrast, BP and CO responses to CPT and IHE/PEI were normal (i.e., increased from baseline). Upon closer inspection, it appears that the increase in BP was achieved by an increase in CO, rather than an increase in TPR. This theory is supported by a concomitant increase in LF/HF measures of HRV during CPT (from 184.4 to 333.6), suggesting an increase in cardiac sympathetic activity combined with vagal withdrawal. These findings are illustrated in Tables 1 and 2.

**Table 1:** Muscle sympathetic nerve activity during CPT and IHE/PEI

	Burst incidence (bursts/100 heart beats)	% of Baseline	Burst frequency (bursts/minute)	% of Baseline	Burst area per minute	% of Baseline
Cold pressor test						
Baseline	47	100	29	100	1.77	100
CPT Minute 1	37	78	27	93	1.53	86
CPT Minute 2	19	40	15	52	0.67	38
CPT Minute 3	23	49	15	52	0.80	45
Handgrip exercise						
Baseline	51	100	28	100	1.98	100
IHE Minute 1	29	57	23	82	0.32	67
IHE Minute 2	23	45	19	68	0.78	39
PEI Minute 1	35	69	25	89	1.32	67
PEI Minute 2	32	63	23	82	1.28	65

Data are reported as 1‐min averages. CPT, cold pressor test; IHE, ischemic handgrip exercise; PEI, postexercise ischemia.

**Table 2:** Hemodynamic response to CPT and IHE/PEI

	Cardiac output (L/min)	Systolic BP (mmHg)	Diastolic BP (mmHg)	Total peripheral resistance (mmHg*min/L)
Cold pressor test				
Baseline	3.9	126.1	71.6	23.8
CPT	4.9	170.5	93.2	25.2
Handgrip exercise				
Baseline	3.8	131.9	72.6	25.3
IHE	5.0	158.9	88.3	22.1
PEI	5.4	163.6	85.9	21.9

Data are reported as 1‐min (Baseline), 2‐min (IHE, PEI), or 3‐min averages (CPT). CPT, cold pressor test; IHE, ischemic handgrip exercise; PEI, postexercise ischemia; BP, blood pressure.

## Discussion

Targeting the CBs for surgical resection or ablation has emerged as a possible therapeutic strategy to mitigate the increased sympathetic activity in multiple disease states such as heart failure and hypertension. Several studies have already emerged with the aim of prospectively evaluating CB resection in the management of heart failure and hypertension (Niewinski et al. 2013; Narkiewicz et al. 2016). However, the existing literature on CB resection in humans is heterogeneous and lacks a comprehensive approach to evaluating the physiological consequences of CB resection in healthy and diseased populations.

Our patient did not experience any clinical consequences of her surgery. After her resection, she had a few episodes of mild orthostasis but had no syncopal events. She leads an active lifestyle and her BP, HR, and baseline oxygenation status remain normal. She has no evidence of sleep disordered breathing or arrhythmia. Her normotension is consistent with previous studies in which BP does not seem to be altered after unilateral CB resection in previously normotensive patients (Dehn and Angell‐James 1987).

Interestingly, our patient showed a large decrease in MSNA in response to traditionally sympathoexcitatory stressors. In intact healthy humans (including postmenopausal women), CPT and IHE/PEI typically result in an increase in HR, BP, and MSNA (Shoemaker et al. 1985; Stickland et al. 2008; Okada et al. 2016). In our patient, both CPT and IHE/PEI caused a decrease in MSNA from baseline values and remained low until the stressor was withdrawn. Prior studies have shown variable but often transient decreases in baseline/resting MSNA values after unilateral CB resection (Stickland et al. 2008; Niewinski et al. 2013; Narkiewicz et al. 2016). Unfortunately, these studies measured MSNA at rest and did not investigate MSNA response to stressors. Although we were not able to obtain preoperative measures of MSNA in our patient, it is intriguing that she demonstrated a relatively robust decrease in MSNA during CPT and IHE/PEI more than 4 years after her CB resection. Thus, although prior studies seem to indicate a relative normalization of resting MSNA over time, these studies overlook what may be a deficiency in sympathoexcitatory response to stress. Ideally, future studies in this area would evaluate baseline and response measures of MSNA pre‐ and postoperatively.

Furthermore, the normal increase in HR and BP during CPT and IHE/PEI despite attenuated MSNA responses may indicate a greater reliance on central rather than peripheral modulation of sympathetic output (i.e., increase in CO vs. increase in TPR; Sayed et al. 2016) when the CB is absent, or an adaptive response of the baroreceptors (Somers et al. 1991; McBryde et al. 2013). Indeed, her CO increased by approximately 26% during CPT with a small, likely nonsignificant increase in TPR. Consistent with this, changes in her HRV during CPT (most notably an increase in LF/HF ratio) illustrate intact central sympathetic response to stress. Interestingly, recent investigations into the sympathetic response to mental stress have found that the MSNA response to stress is variable, i.e., not all subjects showed a characteristic increase in MSNA during mental stress (Porkorski et al. 2004). Despite this, increases in HR and BP were universal. It is posited that the loss of the carotid chemoreflex (an important driver of the sympathoexcitatory response (Stickland et al. 2008)) contributes to an attenuated MSNA response, followed by a compensatory increase in CO to maintain BP. An adaptive response of the baroreflex may then contribute to a further reduction in MSNA. Although speculative, we propose baroreflex adaptive function postparaganglioma resection may be due to a change in compliance of the carotid sinus postoperatively, iatrogenic sympathetic denervation, or withdrawl of the inhibitory influence of the carotid body on baroreflex function (Somers et al. 1991; McBryde et al. 2013). The end result may be a sensitized baroreflex. Furthermore, TPR is maintained despite the reduction in MSNA, likely secondary to the increase in CO along with contributions from other compensatory mechanisms such as the renin–angiotensin system. We hypothesize this feedback loop explains the physiologic changes we saw in our patient, though alternative mechanisms may also play a role.

In our patient, minute ventilation was inversely correlated with S_p_O_2_ during the HVR test. With this, her ventilatory response was at the lower end of the normal range (−0.23 L/min/%) when compared to prior data from subjects with intact CBs (−0.19 to −0.93 L/min/% (Limberg et al. 2016)). It is unlikely this is the result of her postmenopausal status, given women appear to maintain HVR with aging (Pokorski and Marczak 2003; Porkorski et al. 2004). Rather, this indicates carotid body chemosensitivity to hypoxia remained largely intact, though perhaps slightly lower than expected secondary to a relatively short time between unilateral resection and testing. Prior studies have demonstrated that patients with chronic obstructive pulmonary disease have slightly decreased HVR after unilateral CB resection (Honda et al. 1979). In contrast, bilateral CB resection in healthy patients and those with chronic hypoxia may cause permanent abolition of HVR (Timmers et al. 2003; Niewinski et al. 2013). As a consequence, these patients may experience significant ventilatory dysfunction after bilateral CB resection, outweighing any possible benefit of decreased sympathoexcitatory output (Timmers et al. 2003; Paton et al. 2013).

Considerations: While these findings are informative about the complex relationships between the various interactions of the autonomic nervous system, we recognize they come with several limitations. First, these are the inherent considerations given to all single patient case reports. Additionally, our patient was brought in for a single experimental protocol which was not repeated. Lastly, our patient had bilateral paragangliomas, of which only one was resected. It is possible the residual tumor had an effect on her remaining carotid body function.

In summary, we found that in a previously healthy female who underwent unilateral CB resection, HVR and BRS were largely maintained. Despite a typical increase in HR and BP in response to sympathetic stress, MSNA decreased; however, this was accompanied by an increase in CO and the LF/HF ratio of HRV. This case illustrates the complexity of autonomic control as it relates to the CB. While CBs remain a tempting target for therapeutic intervention in multiple common and devastating diseases, more robust descriptive and mechanistic studies are required and a thorough understanding of the physiology of any proposed intervention is needed.

## Conflict of Interest

None.

## References

[phy213212-bib-0001] Dehn, T. C. , and J. E. Angell‐James . 1987 Long‐term effect of carotid endarterectomy on carotid sinus baroreceptor function and blood pressure control. Br. J. Surg. 74:997–1000. 3690247 10.1002/bjs.1800741113 PMC11432596

[phy213212-bib-0002] Fudim, M. , K. L. Groom , C. L. Laffer , J. L. Netterville , D. Robertson , and F. Elijovich . 2015 Effects of carotid body tumor resection on the blood pressure of essential hypertensive patients. J. Am. Soc. Hypertens. 9:435–442. 26051925 10.1016/j.jash.2015.03.006 PMC4785596

[phy213212-bib-0003] Hachiya, T. , I. Hashimoto , M. Saito , and A. P. Blaber . 2012 Peripheral vascular responses of men and women to LBNP. Aviat. Space Environ. Med. 83:118–124. 22303590 10.3357/asem.3174.2012

[phy213212-bib-0004] Honda, Y. , S. Watanabe , I. Hashizume , Y. Satomura , N. Hata , Y. Sakakibara , et al. 1979 Hypoxic chemosensitivity in asthmatic patients two decades after carotid body resection. J. Appl. Physiol. Respir. Environ. Exerc. Physiol. 46:632–638. 457538 10.1152/jappl.1979.46.4.632

[phy213212-bib-0005] Johnson, B. D. , van Helmond N. , T. B. Curry , van Buskirk C. M. , V. A. Convertino , and M. J. Joyner . 2014 Reductions in central venous pressure by lower body negative pressure or blood loss elicit similar hemodynamic responses. J. Appl. Physiol. (1985) 117:131–141. 24876357 10.1152/japplphysiol.00070.2014 PMC4459917

[phy213212-bib-0006] Limberg, J. K. , J. L. Taylor , M. T. Mozer , S. Dube , A. Basu , R. Basu , et al. 2015 Effect of bilateral carotid body resection on cardiac baroreflex control of blood pressure during hypoglycemia. Hypertension 65:1365–1371. 25870188 10.1161/HYPERTENSIONAHA.115.05325 PMC4507506

[phy213212-bib-0007] Limberg, J.K. , B. D. Johnson , W. W. Holbein , S. M. Ranadive , M. T. Mozer , and M. J. Joyner . 2016 Interindividual variability in the dose‐specific effect of dopamine on carotid chemoreceptor sensitivity to hypoxia. J Appl Physiol (1985) 120:138–147. 26586909 10.1152/japplphysiol.00723.2015 PMC4719057

[phy213212-bib-0008] McBryde, F. D. , A. P. Abdala , E. B. Hendy , W. Pijacka , P. Marvar , D. J. Moraes , et al. 2013 The carotid body as a putative therapeutic target for the treatment of neurogenic hypertension. Nat. Commun. 4:2395. 24002774 10.1038/ncomms3395

[phy213212-bib-0009] Melcher, A. , and D. E. Donald . 1981 Maintained ability of carotid baroreflex to regulate arterial pressure during exercise. Am. J. Physiol. (1981) 241:H838–H849. 10.1152/ajpheart.1981.241.6.H838 7325252

[phy213212-bib-0010] Narkiewicz, K. , L. E. Ratcliffe , E. C. Hart , L. J. Briant , M. Chrostowska , J. Wolf , et al. 2016 Unilateral carotid body resection in resistant hypertension: a safety and feasibility trial. JACC Basic Transl. Sci. 1:313–324. 27766316 10.1016/j.jacbts.2016.06.004 PMC5063532

[phy213212-bib-0011] Niewinski, P. , D. Janczak , A. Rucinski , P. Jazwiec , P. A. Sobotka , Z. J. Engelman , et al. 2013 Carotid body removal for treatment of chronic systolic heart failure. Int. J. Cardiol. 168:2506–2509. 23541331 10.1016/j.ijcard.2013.03.011

[phy213212-bib-0012] Okada, Y. , S. S. Jarvis , S. A. Best , J. G. Edwards , J. M. Hendrix , B. Adams‐Huet , et al. 2016 Sympathetic neural and hemodynamic responses during cold pressor test in elderly blacks and whites. Hypertension 67:951–958. 27021009 10.1161/HYPERTENSIONAHA.115.06700 PMC4833636

[phy213212-bib-0013] Paton, J. F. , P. A. Sobotka , M. Fudim , Z. J. Engelman , E. C. Hart , F. D. McBryde , et al. 2013 The carotid body as a therapeutic target for the treatment of sympathetically mediated diseases. Hypertension 61:5–13. 23172927 10.1161/HYPERTENSIONAHA.111.00064

[phy213212-bib-0014] Pokorski, M. , and M. Marczak . 2003 Ventilatory response to hypoxia in elderly women. Ann. Hum. Biol. 30:53–64. 12519654 10.1080/03014460210162000

[phy213212-bib-0015] Porkorski, M. , M. Walski , A. Dymecka , and M. Marczak . 2004 The aging carotid body. J. Physiol. Pharmacol. 55(Suppl 3):107–113. 15611601

[phy213212-bib-0016] Sayed, K. E. , V. G. Macefield , S. L. Hissen , M. J. Joyner , and C. E. Taylor . 2016 Rate of rise in diastolic blood pressure influences vascular sympathetic response to mental stress. J. Physiol. 594:7465–7482. 27690366 10.1113/JP272963 PMC5157061

[phy213212-bib-0017] Shoemaker, J. K. , L. Mattar , P. Kerbeci , S. Trotter , P. Arbeille , and R. L. Hughson . 1985 Wise 2005: stroke volume changes contribute to the pressor response during ischemic handgrip exercise in women. J. Appl. Physiol. 103:228–233. 10.1152/japplphysiol.01334.2006 17412786

[phy213212-bib-0018] Somers, V. K. , A. L. Mark , and F. M. Abboud . 1991 Interaction of baroreceptor and chemoreceptor reflex control of sympathetic nerve activity in normal humans. J. Clin. Invest. 87:1953–1957. 2040688 10.1172/JCI115221 PMC296947

[phy213212-bib-0019] Stickland, M. K. , B. J. Morgan , and J. A. Dempsey . 2008 Carotid chemoreceptor modulation of sympathetic vasoconstrictor outflow during exercise in healthy humans. J. Physiol. 586:1743–1754. 18202096 10.1113/jphysiol.2007.147421 PMC2375684

[phy213212-bib-0020] Thompson, C. A. , D. L. Tatro , D. A. Ludwig , and V. A. Convertino . 1990 Baroreflex responses to acute changes in blood volume in humans. Am. J. Physiol. 259:R792–R798. 2221146 10.1152/ajpregu.1990.259.4.R792

[phy213212-bib-0021] Timmers, H. J. , W. Wieling , J. M. Karemaker , and J. W. Lenders . 2003 Denervation of carotid baro‐ and chemoreceptors in humans. J. Physiol. 553:3–11. 14528027 10.1113/jphysiol.2003.052415 PMC2343492

[phy213212-bib-0022] Victor, R. G. , D. R. Seals , and A. L. Mark . 1987 Differential control of heart rate and sympathetic nerve activity during dynamic exercise. Insight from intraneural recordings in humans. J. Clin. Invest. 79:508–516. 3805279 10.1172/JCI112841 PMC424115

